# Development of a Clinical Interface for a Novel Newborn Resuscitation Device: Human Factors Approach to Understanding Cognitive User Requirements

**DOI:** 10.2196/12055

**Published:** 2019-06-08

**Authors:** Laura Pickup, Alexandra Lang, Lara Shipley, Caroline Henry, James Carpenter, Damon McCartney, Matthew Butler, Barrie Hayes-Gill, Don Sharkey

**Affiliations:** 1 LP Human Factors Ltd Taunton United Kingdom; 2 Trent Simulation and Clinical Skills Centre Nottingham Universities Hospitals NHS Trust Queen's Medical Centre Nottingham United Kingdom; 3 Division of Child Health, Obstetrics and Gynaecology School of Medicine & Health Sciences University of Nottingham Nottingham United Kingdom; 4 SurePulse Medical Limited Medicity Nottingham United Kingdom; 5 Optics and Photonics Research Group Department of Electrical and Electronic Engineering University of Nottingham Nottingham United Kingdom

**Keywords:** neonatal resuscitation, medical device, human factors, user-centred design, applied cognitive task analysis

## Abstract

**Background:**

A novel medical device has been developed to address an unmet need of standardizing and facilitating heart rate recording during neonatal resuscitation. In a time-critical emergency resuscitation, where failure can mean death of an infant, it is vital that clinicians are provided with information in a timely, precise, and clear manner to capacitate appropriate decision making. This new technology provides a hands-free, wireless heart rate monitoring solution that easily fits the clinical pathway and procedure for neonatal resuscitation.

**Objective:**

This study aimed to understand the requirements of the interface design for a new device by using a human factors approach. This approach combined a traditional user-centered design approach with an applied cognitive task analysis to understand the tasks involved, the cognitive requirements, and the potential for error during a neonatal resuscitation scenario.

**Methods:**

Fourteen clinical staff were involved in producing the final design requirements. Two pediatric doctors supported the development of a visual representation of the activities associated with neonatal resuscitation. This design was used to develop a scenario-based workshop. Two workshops were carried out in parallel and involved three pediatric doctors, three neonatal nurses, two advance neonatal practitioners, and four midwives. Both groups came together at the end to reflect on the findings from the separate sessions.

**Results:**

The outputs of this study have provided a comprehensive description of information requirements during neonatal resuscitation and enabled product developers to understand the preferred requirements of the user interface design for the device. The study raised three key areas for the designers to consider, which had not previously been highlighted: (1) interface layout and information priority, as heart rate should be central and occupy two-thirds of the screen; (2) size and portability, to enable positioning of the product local to the baby’s head and allow visibility from all angles; and (3) auditory feedback, to support visual information on heart rate rhythm and reliability of the trace with an early alert for intervention while avoiding parental distress.

**Conclusions:**

This study demonstrates the application of human factors and the applied cognitive task analysis method, which identified previously unidentified user requirements. This methodology provides a useful approach to aid development of the clinical interface for medical devices.

## Introduction

### Background

Globally, there are approximately 3.6 million neonatal deaths annually (ie, in the first 28 days of life), with 70% occurring on the first day of life [1]. Up to 10% of newborns (79,000/year in the United Kingdom and 13 million/year worldwide) require some form of resuscitation at birth, with an estimated 7 million babies worldwide requiring more advanced resuscitation [2]. The correct structured management of resuscitation in the first few “golden” minutes after birth is critical to prevent significant morbidity (eg, cerebral palsy due to hypoxia) or death. There is strong evidence that standardized resuscitation training and algorithms significantly improve newborn outcomes and could reduce mortality by up to 30% [2,3].

International newborn resuscitation guidelines highlight the importance of using the heart rate (HR) to guide resuscitation and stabilization methods [4]. However, many of the methods used to measure HR are inaccurate or technically challenging, particularly in premature infants.

When assessing the HR, practitioners always have access to a stethoscope but use other technologies less frequently [5]. HR assessment using the stethoscope, through auscultation, is inaccurate in about one-third of cases [6,7] and is not continuous; therefore, it needs to be performed every 30 seconds. As such, it is time consuming, which pauses resuscitation and can lead to errors.

This paper describes an enquiry investigating the design and use of a novel medical device developed to address the unmet need of standardizing and facilitating neonatal resuscitation. In an emergency time-critical resuscitation situation where failure can mean death of an infant, it is vital that clinicians are provided information in a timely, precise, and clear manner to support decision making. The nature of this context requires an interface that can ensure both the efficiency and reliability of staff to access the most critical information. A touchscreen interface was considered to be the best hardware solution. The work described here focuses on the development of the design requirements for a touchscreen interface that is integral to this novel medical device.

To understand the requirements of this new device, and specifically, the contributors to interface design, a human factors approach was implemented, which combined a traditional user-centered design approach with an applied cognitive task analysis (ACTA) [8]. The aim of this study was to understand not only the tasks involved but also the cognitive requirements of clinicians. This study has enabled the generation of an interface specification. In addition, the study’s findings provide points of learning to other medical device developers and clinicians, with an aim of understanding the complex requirements and information needs of clinicians during neonatal resuscitation.

### Medical Devices to Measure Heart Rate in Neonates

Other common techniques for monitoring HR in the neonatal intensive care unit, such as electrocardiography or pulse oximetry, were not developed for resuscitation at birth. These systems are used less due to their reliability, delay in HR readings, and practical issues (eg, difficulty ensuring adhesion to the skin) [5,9].

In the delivery room, electrocardiography and pulse oximetry sensors are connected to the main monitors by cables. This can make attachment more challenging and risks cold exposure with the potential for hypothermia, which is an independent risk factor for death in premature babies [10]. Current resuscitation guidance for premature babies highlights the prevention of hypothermia, and therefore, priority is given to drying the baby’s head, putting on a hat, and placing the body (wet) in a plastic bag/wrap [11-13].

To address the issues described, a novel HR monitoring hardware solution using reflectance mode optical photoplethysmograpy, an optical sensor, has been designed. This monitor has been integrated into a single-use newborn hat, specifically for use in newborn babies requiring resuscitation. This solution aims to fit naturally into the existing care pathway, allowing wireless, hands-free, quick, continuous, and accurate HR monitoring via a touchscreen interface as well as minimizing the risk of hypothermia. The effectiveness of the solution is a combination of two features: the forehead placement, where blood flow is preserved even in babies with a low HR (the forehead blood supply comes from the carotid arteries that supply the brain), and the sensor’s patented optical arrangement and signal processing scheme, which has been proven to provide high signal quality from neonatal patients [14]. Additionally, the hat uses wireless communication, allowing greater flexibility in deployment than cable-based solutions.

### Human Factors/Ergonomics in Medical Device Design

The value of human factors/ergonomics (HFE) integration to medical device design and patient safety has been recognized over recent years [15-19], has gained formal recognition in standards [20-23], and is a requirement of the European Medical Devices Directive 93/42 and its 2007 amendment for obtaining Conformité Européenne approval. Concerns still remain about the quality and effectiveness of the interpretation of all relevant standards and integration of HFE within the design/development process. There appears to be a lack of “exemplar case studies” to illustrate how the design process and user-centered design can contribute to product design in health care and how HFE should be routinely implemented [24-26]. This is acknowledged with specific barriers identified within small and medium enterprises such as university spin-out companies [18].

This study contributes to the body of evidence on the application of HFE methods to the formative evaluation of this novel medical device, as required by the relevant standards [23], through a collaboration between a university spin-out company, the School of Medicine, and the Human Factors Research group in the University of Nottingham.

This study focuses specifically on understanding human-computer interactions and user requirements for the computer interface of the device. The aims were (1) to identify gaps in existing knowledge on user requirements for the interface design of a novel resuscitation device and (2) to represent the key design requirements to promote usability of the touchscreen interface of the device.

## Methods

This study collected and analyzed data from intended and representative future users of the new device.

The ACTA method was selected for this study, as it is known to be beneficial to health care domains [8,26]. The ACTA facilitates the elicitation of cognitive requirements from clinicians relative to a particular task and translates them into design requirements for system designers [8]. The ACTA has four key stages. Table 1 highlights how each stage of the method is relevant to understanding the task of neonatal resuscitation. For the purposes of this study, the ACTA method was modified to accommodate clinical working practices, and the simulation interview took the form of an interactive scenario-based workshop.

The workshop aimed to recruit a range of health care professionals, with varying levels of experience and representative of those who might have involvement in neonatal resuscitation procedures. A convenience sampling approach was adopted for the recruitment of participants from two large tertiary-based teaching hospitals in the United Kingdom. Posters and flyers advertised the details of the study, and 12 staff with experience of neonatal resuscitation were successfully recruited (Table 2).

To explore the cognitive requirements further and elicit insight from all practitioners, the workshop protocol divided the practitioners into two groups of six people, split evenly to ensure equal numbers of each job role for each group with the exception of the clinical educator and trainee who were put in different groups. This allowed different levels of experience and job roles to explore the same simulation (Textbox 1). Ethical approval was provided by the University of Nottingham, and all participants gave their informed consent.

The two researchers (LP and AL) familiarized themselves with the task of neonatal resuscitation by observing videos of a simulated resuscitation provided by the two subject matter experts (SMEs; LS and CH who are neonatal doctors with 8 years of resuscitation experience) and follow-up interviews to clarify points of uncertainty and task identification. This was necessary for practical reasons, as the observation of such events cannot be planned. A review of the national neonatal resuscitation algorithm [11] provided the researchers with an understanding of the current UK practice. Finally, relevant international and British standards [20-23] were consulted to provide direction for the designers on medical device recommendations.

There were five outputs from this study that were achieved through the products listed in Table 1:

1. A high-level representation of the tasks required to identify the need and completion of neonatal resuscitation (Table 1 - Stage 1 - task diagram
2. Identification of the key/difficult cognitive requirements for neonatal resuscitation tasks, critical information, and decision points (Table 1 - task diagram and knowledge audit interview)
3. Analysis of the cognitive demand associated with key tasks and potential errors (Table 1 - knowledge audit and simulation interview)
4. User opinion on interface design options to support cognitive requirements, reduce potential for error, and record neonatal resuscitation events (Table 1 - simulation interview)
5. A comprehensive outline of user and design requirements for the interface design and relevant standards (Table 1 - simulation interview and cognitive demands table)

**Table 1:** Description of the applied cognitive task analysis.

Description	Stage 1 - Task diagram	Stage 2 - Knowledge audit interview	Stage 3 - Simulation interview - workshop	Stage 4 - Cognitive demands table
Method	Interviews with two SMEs^a^ familiar with the task of neonatal resuscitation Interview 1: Task identification with SME 1 (150 minutes) Interview 2: Verification of task representation with SME 2 and identification of key/difficult cognitive tasks with SMEs 1 and 2 (75 minutes)	Interview with 2 SMEs (180 minutes). Starting with the use of the knowledge audit probes (Multimedia Appendix 1) to elicit general domain knowledge of how an expert may deal with a neonatal resuscitation while exploring potential errors that novice users may make. Specific examples of how certain cues and strategies supported individual tasks were also explored.	Observation of a challenging scenario (Textbox 1) involving the task of neonatal resuscitation. Each key task is queried to explore the critical cues, assessment, actions, and potential for error: What actions, if any, would you take at this point? What do you think is going on here? What is your assessment of the situation at this point in time? What pieces of information led you to this situation assessment and these actions? What errors would an inexperienced person be likely to make in this situation?	To summarize and integrate the information obtained from the previous three steps and interview data gathered prior to the study.
Purpose	To provide a broad view of the task and identify difficult cognitive components	To highlight which aspects of the task require expertise and which cues and strategies are relied upon to understand the impact on the novice user	To determine the cognitive process involved with key tasks and potential error	A comprehensive record of the findings of the project goals
Products	Key tasks associated with neonatal resuscitation using sticky notes (Figure 1) Visual representation tasks using Microsoft Visio software (version 2013) Verification of task representation Key/difficult cognitive tasks (eg, those requiring decision making, judgements, assessments, or problem solving)	Identification of critical cues and interpretation of information to diagnose and predict situation Identification of strategies relied upon by expert users Identification of the potential for errors in novice users	Identification of difficult cognitive components of task, information, and priorities Identification of critical cues relevant to decision making for each key task and potential for errors in novice users Essential and desirable information requirements Group mock-up of interface design on a cardboard model Individual annotation of a paper-based image of the intended interface screen	A spreadsheet of the data collated through the study

^a^SME: subject matter expert.

**Table 2:** Details of the workshop participants.

Job role	Number of years/range of experience	Number of participants
Neonatal trainee nurse	2	1
Neonatal nurse	1-16	2
Midwife	0-25	4
Pediatric/neonatal doctor	1.5-5	2
Neonatal clinical nurse educator	30	1
Advanced neonatal nurse practitioner	15-22	2

**Simulation of a challenging scenario used to probe practitioners during the workshop.**

**Past Clinical History**

A first-time mother at 42 weeks’ gestation presents with her baby stuck due to shoulder dystocia. She has a slight fever and no past medical history but has received diamorphine during her labor. Labor was induced through artificial rupture of the membrane. She has prolonged rupture of membranes and labor has been ongoing for 24 hours. The baby’s head was delivered 10 minutes before the baby’s body and the airway appears clear.

1. **Assessment of observations**
  - Baby presents floppy, white, and not responding to vigorous stimulus. Heart rate < 60 beats/minute with stethoscope and no respiratory effort evident.
2. **Progression of intervention**
  - After two sets of five inflation breaths, there is still no chest movement.
3. **Chest compressions commenced**
  - The chest moved but heart rate remained slow.

**Figure 1:**
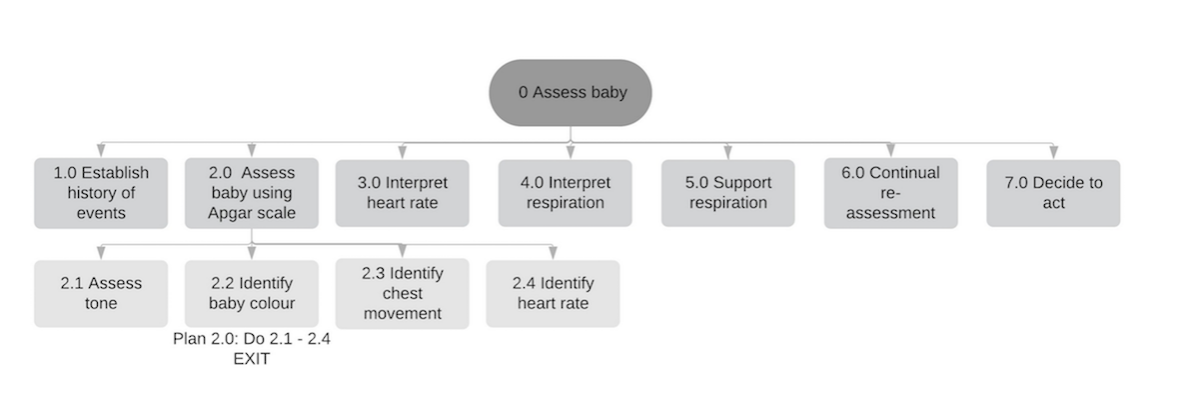
A sample of the representation of the task analysis completed with clinicians to illustrate neonatal resuscitation.

## Results

The practitioner workshop involved pediatric doctors (n=2), neonatal nurses and educators (n=3), advanced neonatal nurse practitioners (n=2), and midwives (n=4) with 0-30 years of experience (average of 11 years). The workshop and SME interviews identified factors relevant to device and interface design not previously considered by the design team.

### Context of Neonatal Resuscitation Tasks

The critical characteristics of a neonatal resuscitation were described by practitioners as time pressured and unpredictable albeit well-rehearsed. This is likely to be an emotional and stressful situation for the parents involved. The location of a neonatal resuscitation may vary and could include the labor suite, midwife-led unit, birthing pool room, operating theatre, patient’s home, or ambulance. Participants suggested that the portability of the system should therefore be prioritized. Practitioners considered the attachment features of the device to replicate those of a car satellite navigation screen, with options to secure (which implies the device is attached but can be adjusted) and rotate the screen to ensure a continual good line of sight. The physical environment suggests lighting may also vary (eg, bright theatre lights and variability in lighting within a single resuscitation). The device may also remain in use when transferring the baby between delivery and the neonatal unit or in ambulances, hence making it resistant to vibration and movement.

Alarms were considered useful only in certain contexts and the type, such as frequency, and pitch of alarm required sensitivity in design to avoid undesirable consequences for parents and clinicians [27,28].

The device may be used in the context of other medical devices (eg, Resuscitaire, a portable “platform” for neonatal resuscitation integrated with required equipment). Compatibility between equipment is essential to ensure usability and reliability.

### Tasks Relevant to Neonatal Resuscitation

The SME interviews (interviews 1 and 2 in Table 1) suggested seven relevant high-level cognitive tasks and produced the first study output, a high-level representation of the tasks required during neonatal resuscitation (Multimedia Appendix 2): establish a history of events, assess baby, interpret HR, interpret respiration, support respiration, continual reassessment, and decide to act.

These tasks were considered based on the core principles of the task analysis [29]. The top layer represents “what” has to be done (Multimedia Appendix 2), and further descriptions in the layer below describe “how” it has to be done (Multimedia Appendix 2). A visual representation was shared with clinicians within the workshops, and a consensus was reached for the final presentation. Figure 1 illustrates a sample of the representation shared.

Nine tasks in total were agreed upon by the SMEs and workshop participants, to have a cognitive element to them (Multimedia Appendix 2). The nine tasks included receive antenatal history, assess baby using Apgar score [30], interpret chest movement, interpret HR, decide on action, direct view and clearing of airway, assist breathing, decide to intubate, and decide to medicate.

### Cognitive Requirements, Demands, and Potential Error

The interviews completed during the development of the task diagram and the knowledge audit interview elicited information relevant to the difficulty and nature of the cognitive work including cues, assessment, judgements, problem solving, decisions, and actions combined with potential challenges and errors and strategies relevant to the nine cognitive tasks.

The findings from these first two stages of the ACTA method were verified and enriched by data obtained during the simulation interview workshop. The data from all three stages of the ACTA method were collated within a spreadsheet (a template of the one used is provided in Multimedia Appendix 2) and then combined and simplified to produce a cognitive demand table for each of the nine cognitive tasks (Textbox 2). These created the second and third study outputs.

**Cognitive demand table to assess the baby using the Apgar score to inform decision and actions.**

**Why is it difficult?**

- Interpretation of heart rate and chest movement relative to normal parameters
- Judgement of accuracy and reliability of heart rate display
- Reliance on previous experience and recognition of “normal” heart rate and chest movement to inform decision and actions
- Multiple tasks in short time frame: visual check of heart rate, chest movement, tone, and skin color. Continual re-evaluation every 30 seconds
- Requires expertise to ensure decision making within a short timeframe and potentially stressful environment

**Common errors**

- Accuracy in interpretation of heart rate
- Fail to recall normal heart rate and chest movement
- Estimation/recall of time elapsed between key events
- Failure to recognize when to act (eg, call for help and intubation)
- Avoidance behavior: fear to act/“failure to rescue”
- Overreliance on technology (lacking reliability) and colleague’s earlier assessment
- Quiet breathing missed (eg, preterm babies)
- Lighting can distort baby color

**Cues**

- Absent heart rate, heart rate < 60 beats/minute
- Heart rate > 100 beats/minute
- Floppy
- White coloring
- No breathing/gasping
- Stressful environment
- Absence of baby crying

**Strategies**

- Consider how to obtain support with minimal alarm to parent
- Continually question interpretation/reliability of information
- Continual reassessment at 30 seconds and after 1, 5, and 10 minutes
- Closer inspection (eg, ear to mouth), observe rib cage and abdomen, listen for absence of sound or gasping

**Figure 2:**
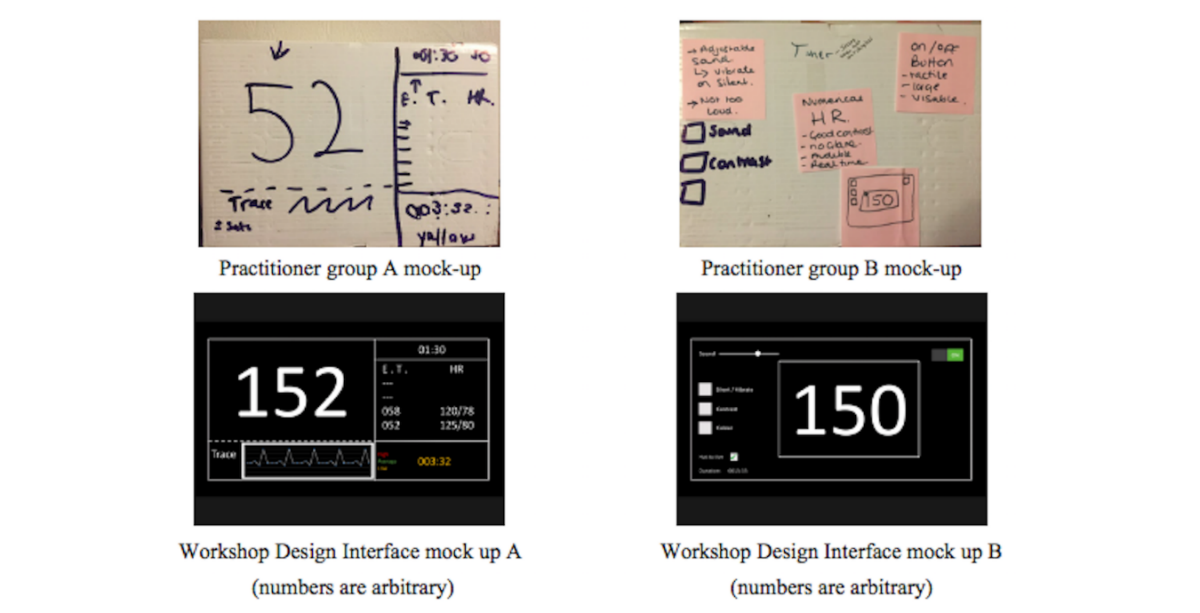
Mock-up of interface designs produced during simulation interview.

The two workshop groups further informed the fourth and final study outputs, which produced a specification and illustrated mock ups with notable differences in the priorities for design. One group preferred to protect the simplicity of the device and represent HR as the only physiological marker, with an event timeline running in the background. The other group preferred to include oxygen saturation and a visible record of an event timeline (Figure 2).

**Figure 3:**
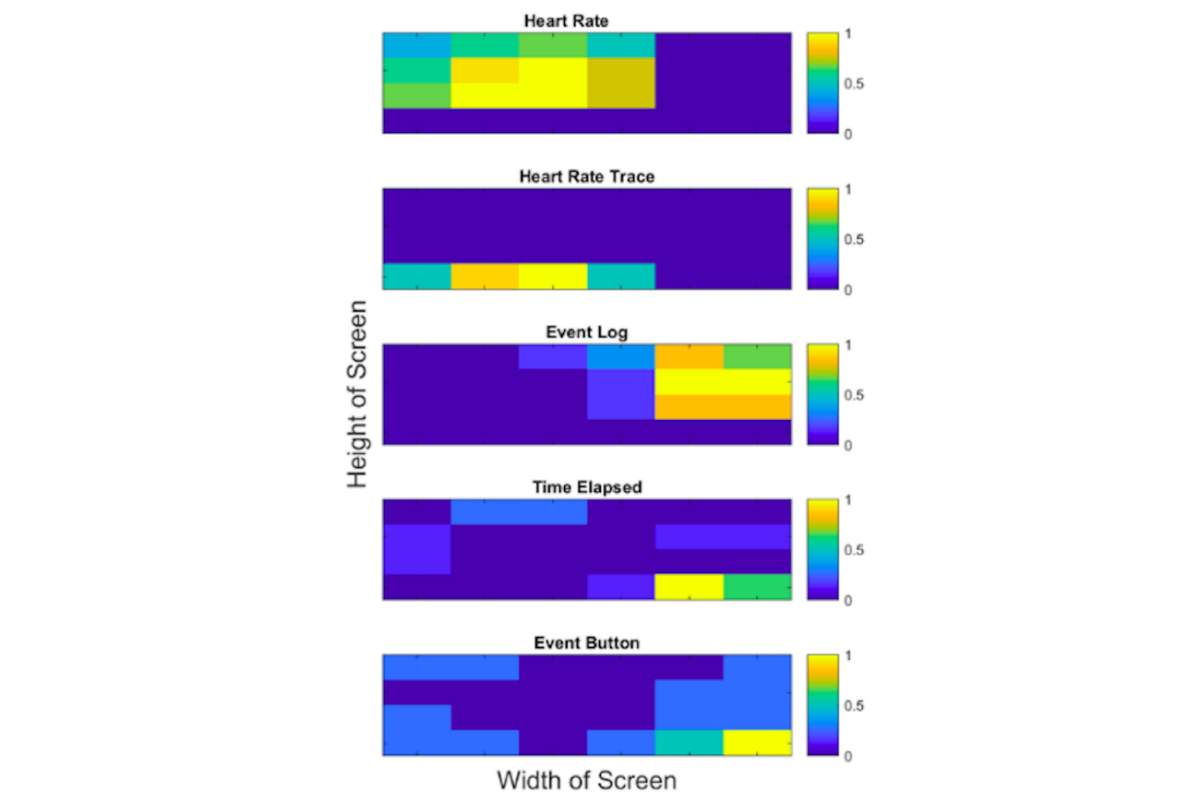
Heat map indicating consensus for the location of key information sources.

After the two workshop groups had worked through the simulation independently, they together presented their specifications and mock ups. Individual participants were then asked to reflect on the work they had done in their groups and on the presentation from the other groups to produce their own personal interface design. These individual contributions were analyzed to interpret group preferences and produce cumulative representations of the data as a heat map (Figure 3). This indicates consensus on the location of interface information sources, summarizing individual location preferences (12 practitioners) of the five information types. The x-axis indicates the width of the screen and the y-axis indicates the height of the screen. Each screen was broken up into 24 areas (6 along the x-axis and 4 along the y-axis). The color bars are normalized against the maximum number of practitioner votes for each area on the heat map.

In summary, the stages within the ACTA method are provided below:

1. Task diagram
  - A breakdown of the physical and cognitive activities involved in the context of neonatal resuscitation
  - Visual representation of SME perspectives of the key cognitive activities
2. Knowledge audit interviews
  - Detailed descriptions of the nature of the cognitive work required, cues, and strategies relied upon and potential for error
  - Insight into differences between expert and novice practitioners in the context of neonatal resuscitation
  - Examples of previous experiences that revealed influences of the people involved/present, environmental factors, and the emotional nature of the context.
3. Simulation interview (workshop)
  - Verification of the task diagram and understanding of cognitive requirements based on a broader group of experts
  - Additional insights into cognitive work required, cues and strategies relied upon, and potential for error based on the experience of the participants
  - Consideration of future user and health care contexts
  - Design suggestions that reflect practitioners’ preferences and understanding of the cognitive activities and potential errors discussed
4. Cognitive demands table
  - An assimilation of all of the abovementioned findings in a usable format to inform and justify the development of the design requirements

**Figure 4:**
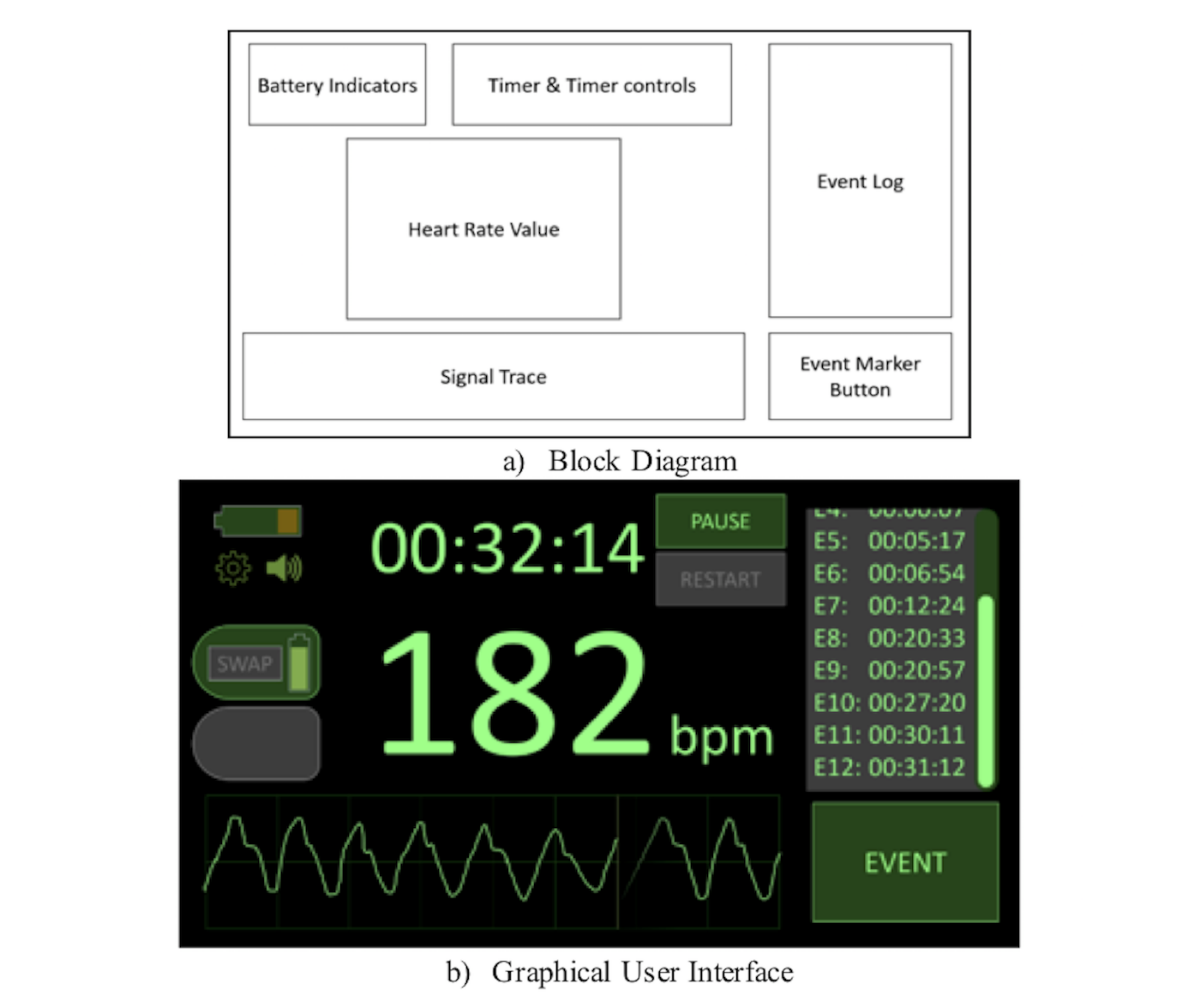
Block diagram (a) and the Graphical User Interface concept (b) developed from the block diagram.

Themes relative to essential and desirable characteristics for the interface were elicited. These were combined with recommendations from international standards to produce a set of design requirements [20,23,31-35]. A typical example of the information contained within these requirements is provided in Textbox 3. The final decision for timer position was influenced by users and optimization of the display screen space. The information obtained informed the final design developed and indicated priorities for future usability testing. This information and the heat map were developed as a block diagram and informed the graphical user interface concept (Figure 4).

**Design requirements related to information layout.**

**Essential characteristics**

Heart rate should be central and the largest text on the screen. This should allow visibility from all angles and the heart rate information should occupy two-thirds of the screen.

**Desirable characteristics**

Divide the screen to have a margin on the side of the screen with buttons to mark events. One group suggested illustration of a visible timeline. The second group did not agree with any more information than essentially required (eg, heart rate).

**International standards and recommendations**

Hierarchy of the content of information displayed should be implied by the layout. The most important information should occupy priority space, typically top left for large screens and central for smaller screens, with adequate blank space and borders to separate information sources

## Discussion

### Principal Findings

To our knowledge, the ACTA method has not been used in the development of resuscitation devices. There are many HFE methods relevant to the design process [36,37]. The value of methods suitable for identifying user requirements was considered previously [17], and that paper concluded that both focus groups and user testing were beneficial. ACTA in medical device design does not appear to be well applied [18] despite recognition that it could be a useful tool in the domain of health care [8]. The application of such methods, rather than just the completion of the traditional hierarchical task analysis, is well recognized for their benefit of formatively understanding critical cues, decision making, judgements, constraints, and potential errors in the context of a work situation; however, they are also considered resource intensive [8,38,36].

The ACTA was developed to address some of these issues. The method was developed to allow practitioners within the area of work studied and system designers to elicit cognitive requirements relative to task performance and translate these into design requirements [8].

This approach was considered desirable for this study as a method to understand user decision making and critical information requirements from the intended interface and to illustrate a method that could be applied by practitioners themselves in future design/evaluation of medical devices. In addition, pragmatically, the time available suggested that efficiency was desirable in any method selected, which the ACTA offered.

This method has allowed the tasks required for neonatal resuscitation to be fully considered in relation to cognitive requirements, actions, and potential errors. This participatory approach has offered a systematic analysis of the resuscitation process, described as “logical and rigorous” by the SMEs. Successful implementation within the context of health care has been suggested as benefiting from such participation to ensure that relevant stakeholders influence the design of an intervention to fit their own contexts [39]. ACTA allowed user requirements to be identified specific to different contexts and stages within the resuscitation process.

**Figure 5:**
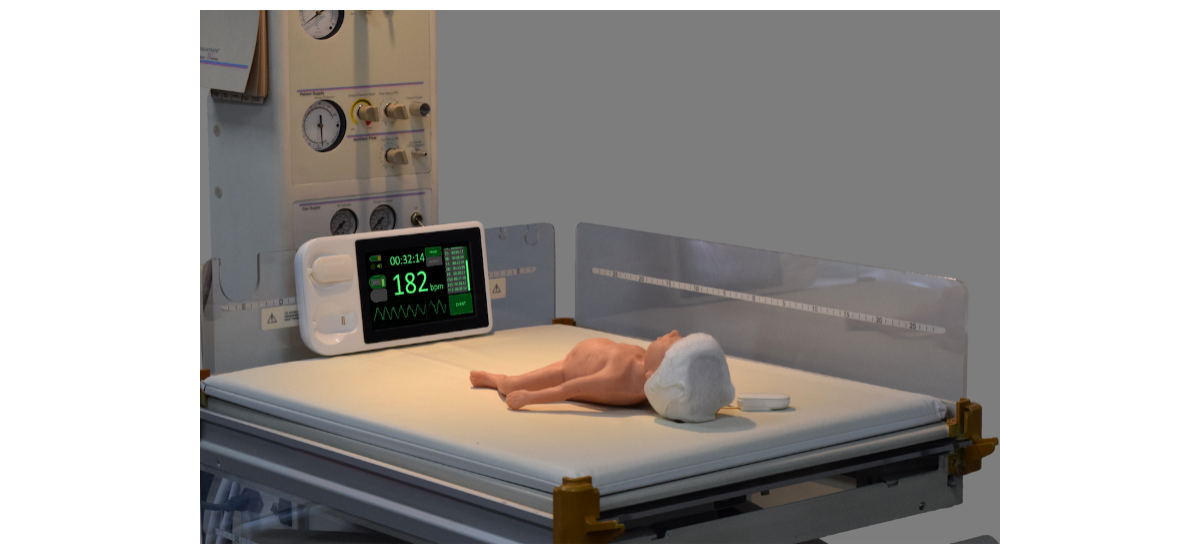
Resuscitation device comprises a single-use thermally insulating hat that communicates wirelessly (via battery powered modules) with a display mounted on the resuscitation table.

The success of the ACTA approach came from engaging participants from different job roles to consider contexts familiar to them and ensure practitioners considered cues with the greatest significance to completing the required tasks, likely errors, and how interface design can support these tasks. The outputs of this study have provided a comprehensive description of information requirements during neonatal resuscitation and enabled product developers to understand the core and preferred requirements of the user interface design for the device. These outputs have been used to develop an interface, which prioritizes simplicity and provides a set of user requirements, to test the device during future testing (Figure 5).

The study raised three key areas for the designers to consider, which had not previously been identified: interface layout and information priority, size and portability of the device, and auditory feedback.

The amount of information, which ultimately influences the size of the screen, will be determined by the intended function of the technology. Considering the task of neonatal resuscitation, it becomes apparent that early on in the process of resuscitation, HR is the key indicator used by practitioners. This information was prioritized by both groups and all individual designs of the interface. Some preferred that this alone should be the function of the device. It was considered desirable to ensure the device had a relatively small interface that could be positioned freely and local to the baby’s head. The auditory feedback proposed by practitioners was to support visual information and interventions early on in the resuscitation process. The nature of the feedback should communicate information on HR, such as rhythm and reliability of the trace. The practitioners went on to suggest that with different auditory settings, the device could be used as a monitoring device within a neonatal unit, not previously considered by the developers. The implications of auditory feedback raised the importance of considering both practitioners and patient representatives (eg, parents within future usability testing) [39].

An integral timer was also considered essential, as it would serve as an indicator for the timeline of events within a resuscitation. Currently, the clock started at the time of birth is integral to the Resuscitaire, but future user testing needs to explore how the novel device will influence this practice.

Considering the community setting, there may be less access to oxygen saturation devices. The novel HR device has the potential to compensate for this absence. Within secondary care, the oxygen saturation devices were considered useful when intubating, to secure an airway for the transfer to a neonatal unit. Including oxygen saturations implied greater usability for the device in alternative situations and work contexts in the future. However, practitioners also acknowledged a risk where oxygen saturations could actually be a distraction for less experienced practitioners from the critical cue of HR, which is considered a better indicator of neonatal status and relied upon by expert practitioners.

The benefit of having an HR reading immediately next to the baby’s head on the wireless module was considered of high value. This would reduce the need to continually look between the baby’s head and an interface screen, where the HR would be viewed at a distance. Suggestions were made about the functionality of the wireless module (Figure 1), including how it could be the component within the device used to download contextual information such as an event log. This would avoid the need for a separate memory stick or disc to store data, reducing the risk of lost device components or information, which is a current problem with other medical devices.

Failure to recognize or acknowledge a deteriorating HR was considered. The device design could incorporate feedback to increase awareness of this critical cue. A change in screen color was suggested as the preferred prompt by some; however, further usability testing is required to find out if this improves practitioner performance in reacting to a deteriorating HR or is perceived as distracting or anxiety provoking. How color is used on the interface will also need to be explored by using color convention guidance and usability testing [20,23,32,33].

The final decisions made on the interface layout were based on the optimization of the screen by the design team. “Future proofing” the device was also considered during the workshop and generated an enthusiastic discussion on how an additional, but linked, mobile device could be used to assist a designated scribe in recording key events within the resuscitation timeline. Currently, the accuracy of a written report of neonatal resuscitation is variable, usually involving the most inexperienced team member to scribe. Future electronic records were proposed as complementary to an event marker control, operated by those delivering or supporting the resuscitation, while recognizing that the timing may be slightly delayed. However, this was considered sufficient to develop a retrospective view of the sequence of events. The electronic recording of these data was considered of significant value for those in governance roles, clinical learning, and audit.

### Strengths and Limitations

The ACTA method provides an efficient, comprehensive, and participatory approach capable of understanding user decision making and critical information requirements from the intended interface. Practitioners discussed the potential for this device beyond the original context considered by the developers.

The limitations of the study were in the sampling of clinicians. Volunteers led to the larger ratio of nurses and midwives to doctors. However, the SMEs were experienced doctors in neonatal resuscitation and fully engaged in the whole study. Limitations of this study have constrained further development of the interface, and device, simulation, and usability testing should ensure the views and suggestions raised by participants can be tested and translated into a real-world device.

### Conclusions

This is the first study to apply the ACTA approach to elicit user requirements for a novel device for neonatal resuscitation. This study demonstrates the application of human factors to inform the development of resuscitation devices, and more generally, for medical device developers and clinicians in the design and evaluation of medical technologies.

The study has provided previously unidentified user requirements and details about the variables, which will inform future usability testing of the interface developed.

## Appendix

Multimedia Appendix 1 Knowledge audit probe.

Multimedia Appendix 2 Cognitive tabular task analysis.

## Abbreviations

ACTA: applied cognitive task analysis
HFE: Human Factors/Ergonomics
HR: heart rate
SME: subject matter expert
