# Supplementary material for: Development of a Clinical Interface for a Novel Newborn Resuscitation Device: Human Factors Approach to Understanding Cognitive User Requirements
Source: JMIR Hum Factors. 2019 Jun 8;6(2):e12055. doi: 10.2196/12055 (PMC6592395; doi:10.2196/12055)
Supplement: Multimedia Appendix 1 [file humanfactors_v6i2e12055_app1.pdf]

Supplementary material Knowledge Audit Probes

Basic Probes

1. **Past and future** – Is there a time when you walked into the middle of a situation and knew exactly how things got there and where they were headed?
2. **Big picture** – can you give me an example of what is important about the big picture for this task? What are the major elements you have to know to keep and keep track of?
3. **Noticing** – have you had experiences where part of a situation just ‘popped’ out at you; where you noticed things going on that others didn’t catch? What is an example?
4. **Job smarts** – when you do this task, are there ways of working smart or accomplishing more with less – that you have found especially useful?
5. **Opportunities/Improvising** – can you think of an example when you have improvised in this task or noticed an opportunity to do something better?
6. **Self-monitoring** – can you think of a time when you realized that you would need to change the way you were performing in order to get the job done?

Optional Probes

1. **Anomalies –** can you describe an instance when you spotted a deviation from the norm, or knew something was amiss?
2. **Equipment difficulties** – have there been times when the equipment pointed in one direction but your own judgement told you to do something else? Or when you had to rely on experience to avoid being led astray by the equipment?

The University of Nottingham
UNITED KINGDOM · CHINA · MALAYSIA

Task data v1.0

**Task ID**<br><br>**Task Description**	**Task cognitive skills:**<br><br>Assessment ☐<br><br>Judgement ☐<br><br>Problem solving ☐<br><br>Decision making ☐
**Action**	**Assessment**
**Critical cues**	**Decision**
**Likely Errors**	**Tricks of the trade**
